# Non-invasive MRI of blood-cerebrospinal fluid-barrier function in a mouse model of Alzheimer’s disease: a potential biomarker of early pathology

**DOI:** 10.1186/s12987-024-00597-7

**Published:** 2024-12-04

**Authors:** Charith Perera, Renata Cruz, Noam Shemesh, Tânia Carvalho, David L. Thomas, Jack Wells, Andrada Ianuș

**Affiliations:** 1 UCL Centre for Advanced Biomedical Imaging, Division of Medicine, University College London, 72 Huntley Street, London, WC1E 6DD UK; 2 Champalimaud Research, Champalimaud Foundation, Av. Brasilia, Lisbon, 1400-038 Portugal; 3 Neuroradiological Academic Unit, Department of Brain Repair and Rehabilitation, UCL Queen Square Institute of Neurology, Queen Square, London, WC1N 3BG UK; 4 Dementia Research Centre, UCL Queen Square Institute of Neurology, Queen Square, London, WC1N 3BG UK; 5 Wellcome Centre for Human Neuroimaging, UCL Queen Square Institute of Neurology, University College London, Queen Square, London, WC1N 3BG UK; 6 King’s College London, School of Biomedical Engineering and Imaging Sciences, Imaging Physics and Engineering Research Department; Cancer Imaging Research Department, St Thomas’ Hospital, Westminster Bridge Road, London, SE1 7EH UK

**Keywords:** Alzheimer’s disease, Choroid plexus, Blood CSF barrier, Brain perfusion, Arterial spin labelling MRI, 3xTg mouse model

## Abstract

**Background:**

Choroid plexus (CP) or blood-cerebrospinal fluid-barrier (BCSFB) is a unique functional tissue which lines the brain’s fluid-filled ventricles, with a crucial role in CSF production and clearance. BCSFB dysfunction is thought to contribute to toxic protein build-up in neurodegenerative disorders, including Alzheimer’s disease (AD). However, the dynamics of this process remain unknown, mainly due to the paucity of in-vivo methods for assessing CP function.

**Methods:**

We harness recent developments in Arterial Spin Labelling MRI to measure water delivery across the BCSFB as a proxy for CP function, as well as cerebral blood flow (CBF), at different stages of AD in the widely used triple transgenic mouse model (3xTg), with ages between 8 and 32 weeks. We further compared the MRI results with Y-maze behaviour testing, and histologically validated the expected pathological changes, which recapitulate both amyloid and tau deposition.

**Results:**

Total BCSFB-mediated water delivery is significantly higher in 3xTg mice (> 50%) from 8 weeks (preclinical stage), an increase which is not explained by differences in ventricular volumes, while tissue parameters such as CBF and T1 are not different between groups at all ages. Behaviour differences between the groups were observed starting at 20 weeks, especially in terms of locomotion, with 3xTg animals showing a significantly smaller number of arm entries in the Y-maze.

**Conclusions:**

Our work strongly suggests the involvement of CP in the early stages of AD, before the onset of symptoms and behavioural changes, providing a potential biomarker of pathology.

**Supplementary Information:**

The online version contains supplementary material available at 10.1186/s12987-024-00597-7.

## Background

The choroid plexus (CP) or blood-cerebrospinal fluid-barrier (BCSFB) is a unique functional tissue that pervades the brain’s fluid-filled ventricles. It is composed of a tight epithelium and stroma fed by a dense network of permeable capillaries, giving rise to a rate of arterial perfusion 5–10× greater than gray matter [1]. As the primary source of cerebrospinal fluid (CSF) replenishment in the central nervous system and a key site of clearance to the circulation [2], healthy BCSFB function is likely to be deeply entwined with the workings of CSF-mediated brain clearance processes such as the glymphatic system [3].

Dysfunction of the CP-CSF-axis is thought to contribute to the build-up of toxic proteins that are one of the hallmarks of Alzheimer’s disease (AD) [4–7], with CSF biomarkers showing promise in the diagnostic workup of AD [8]. Moreover, post-mortem studies [9, 10] and preclinical ex-vivo studies [11] have shown AD-related differences in CP gene expression and morphology. To date, however, our understanding of CP function and its dynamics over the course of the disease has been restricted by a lack of non-invasive measurement techniques meaning that, despite its unique physiological significance, there is relatively little in-vivo data describing CP function in the healthy and diseased brain.

Recently, several imaging studies have taken an interest in mapping CP changes related to AD pathology, studying alterations in various aspects of morphology and function. For instance, in patients with AD, structural MRI data has shown an increase in CP volume with disease severity [12–14], arterial spin labelling (ASL) MRI and dynamic contrast enhanced (DCE) MRI have shown alterations in CP blood flow [14] and permeability to contrast agent, respectively [12], while dynamic PET has revealed a reduction in CSF clearance of tau and amyloid tracers [15–17]. Many of these techniques make use of exogenous tracers that might not necessarily reflect the BCSFB-mediated transport [18] and can have limitations for clinical usage and longitudinal studies. The general principle of ASL is to label a bolus of intravascular blood water using RF pulses (i.e. without exogenous tracers), and subsequently measure the difference between control and labelled images at different inflow times, thereby permitting the non-invasive quantification of rates of water delivery to the tissue as an estimate of tissue perfusion.

To measure CP function in-vivo without using external tracers, a novel MRI approach based on ASL at ultra long echo time was recently proposed [19] to measure the rate of delivery of labelled arterial blood water into ventricular CSF across the BCSFB. To date, this approach has been employed to study changes in BCSFB function in rodent models of ageing [19], pharmacomodulation [20] and systemic hypertension [21]. Recent work has also demonstrated the feasibility of this translational approach to capture the dynamic exchange of labelled blood-water into the CSF in the human brain [22].

Here, we harness ASL MRI to investigate, in-vivo, the alterations of CP function due to AD pathology, its dynamics with disease progression as well as compare it to cerebral perfusion. To this end, we capture MRI measurements of cerebral blood flow and BCSFB water delivery at different disease stages, together with behavioural testing and histopathology analysis in the widely employed triple transgenic mouse model of AD (3xTg), which recapitulates both amyloid and tau pathology. To our knowledge this represents the first investigation of choroid plexus function in AD using non-invasive, translational imaging techniques, which may provide a novel and sensitive biomarker of AD pathology.

## Methods

### Animals

All animal experiments followed ethical and experimental procedures in agreement with Directive 2010/63 of the European Parliament and of the Council, and all the experiments in this study were preapproved by the Champalimaud Animal Welfare Body and the national competent authority (Direcção Geral de Alimentação e Veterinária, DGAV). The AD mouse strain used for this research project, B6;129-Tg(APPSwe, tauP301L)1Lfa *Psen1*^*tm1Mpm*^/Mmjax, RRID: MMRRC_034830-JAX, was obtained from the Mutant Mouse Resource and Research Center (MMRRC) at The Jackson Laboratory, an NIH-funded strain repository, and was donated to the MMRRC by Frank Laferla, Ph.D., University of California, Irvine. Mark P. Mattson, Ph.D., Johns Hopkins University, School of Medicine. For controls, we used hybrid B6129SF2/J mice, i.e. the second filial generation of crossings between C57BL/6J females (B6) and 129S1/SvImJ males (129 S), which are the suggested controls for this particular AD strain.

3xTg mice exhibit pathological and behavioural changes specific to AD, including deposition of extracellular amyloid beta (Aβ) plaques, intracellular neurofibrillary tangles and memory impairment, and are one of the most commonly employed mouse models for AD research [23, 24]. Female 3xTg and normal control (NC) mice were evaluated at 8, 14, 20 and 32 weeks of age to reflect the pre-, sub-, early- and mid-clinical stage of AD, respectively, as performed in a recent ASL study [25]. The evaluation consisted of behavioural testing followed by MRI. In total forty 3xTg and thirty-one NC mice were included in the study (N_TG,8w_ = 10, N_NC,8w_ = 10; N_TG,14w_ = 10, N_NC,14w_ = 7; N_TG,20w_ = 10, N_NC,20w_ = 7; N_TG,32w_ = 10, N_NC,32w_ = 7), following sample sizes previously employed to detect changes in cerebral blood flow based on ASL MRI [25]. Littermates were studied sequentially, while different cages were studied in different sessions when the animals reached the desired ages. The researchers were aware of the group allocation at all stages, nevertheless the experiments and data analysis were performed following the exact same steps for all animals to minimise any possible bias. All mice had free access to food and water and were housed in cages, in the same environmentally controlled room under a 12-h light/dark cycle.

### Behaviour testing

Memory impairment has been observed in rodent models of Alzheimer’s disease through various behaviour tests, which can be used to assess the severity of AD symptoms [26]. To explore possible links between known behaviour deficits of this model and the MRI measurements of cerebral blood flow and BCSFB function, we employed the Y maze spontaneous alternations (SA) test to assess cognitive deficits and short-term memory. The maze has three identical arms which the mice are free to explore. When the animals visit three distinct arms consecutively, the pattern is referred to as a spontaneous alternation. As rodents typically prefer to investigate new environments, a decreased SA score (i.e. the percentage of SA from the total number of arm entries) might reflect a possible impairment in short term memory. Such results have been shown in transgenic 3xTg mice [27].

The Y-maze was built in-house from white matte acrylic with the following dimensions (arm length: 35 cm, width: 5 cm, and height: 15 cm; angle between arms: 120 degrees) [28]. The set-up was placed in a foamed box to improve isolation from external sources of noise and vibration and was equipped with a 30 fpm video camera to record the movement and infrared lights to reduce any stressful elements from the environment [29]. The animals were handled for 20 min a day for 2 days before the behaviour experiments [26]. On the day of the testing, the mice were acclimatised to the experimental room for at least 30 min, after which they were introduced to the maze and left free to explore for a total of 8 min (2 min for habituation and 6 min for testing).

#### Behavioural data analysis

Based on the videos, the sequence of arm entries was manually recorded. An arm entry was considered only if all four limbs of the animal were within the arm and an alternation was counted in the event of consecutive entries into three distinct arms. We recorded the total number of arm entries which reflect the activity and locomotion during testing, and then we calculated the SA score (%SA) as the percentage of spontaneous alternations from the total number of arm entries:

1 $$\:\%SA=\frac{\#\:Spontaneous\:alternations}{\#\:Arm\:entries-2}*100$$

### In-vivo MRI for mapping tissue perfusion

In this study we employed non-invasive ASL MRI to estimate cerebral blood flow (CBF) in three different brain regions - cortex, hippocampus (HC), and midbrain (MB). We map various quantitative parameters, including lateral ventricular volume, BCSFB-mediated total water delivery, tissue CBF and T1 (cortex, hippocampus and midbrain), as well as T1 values of CSF (T1_CSF_).

Images were acquired on a 9.4 T Bruker BioSpec scanner operating an AVANCE III HD console and using a gradient system capable of producing up to 660 mT/m in each direction. An 86 mm volume was used for transmission and ensured a relatively uniform B1 profile, while a 4-element array receive-only cryogenic coil (Bruker BioSpin, Fallanden, Switzerland) was used for signal reception [30].

#### Animal preparation and monitoring

Anaesthesia was induced using 5% isoflurane which was then reduced to 2% during the placement of the animal in an MR compatible bed. The mice were head fixed using ear bars and a bite bar, and eye ointment was applied to prevent dryness. A nose cone was used for constant anaesthesia administration through inhalation and a scavenger pump assured no isoflurane build-up in the scanner. Once inside the scanner, the isoflurane concentration was gradually reduced to 1-1.5%. During the experiments, animals were breathing oxygen-enriched medical air with an O2 concentration of 28%. Their breathing rate was monitored throughout the experiment using a respiration pillow sensor (SA Instruments Inc., Stony Brook, USA) and kept in the range of 70–100 bpm. The mice were placed over a heating pad and their temperature was monitored via an optic fibre rectal temperature probe (SA Instruments, Inc., Stony Brook, New York, USA), and kept at 36 ± 0.5ºC. Each scanning session lasted for a total of 1.5 h.

#### Anatomical reference scans

A T2-TurboRARE sequence (fast-spin echo, Paravision v6.0.1) was applied to acquire sagittal and coronal anatomical reference images to clearly visualise the location of the major CSF compartments in the brains of control and 3xTg mice. T2-TurboRARE sequence parameters were: field of view (FOV) = 20 mm × 16 mm; matrix size = 200 × 160; RARE factor = 8; effective echo time (TE) = 35.61 ms; repetition time (TR) = 2200 ms [20].

Sagittal anatomical reference images (15 × 0.4 mm slices) were used to position the coronal anatomical reference imaging slice and the ASL imaging slices. Coronal anatomical reference images (6 × 0.4 mm slices, 2.4 mm total) were manually positioned to align with the caudal region of the lateral ventricles. Subsequently, these coronal images were manually segmented to provide subject-wise estimates of lateral ventricular volume [31].

Following ventricular measurements, one 32 weeks old 3xTg mouse was excluded due to ventriculomegaly, with a ventricular volume ~ 4 times the group average, which is a potential indicator of a hydrocephalus diagnosis, and thus may present with a ventricular-CSF system which is not representative of AD or healthy control conditions. Following this exclusion, an additional animal was included in the study to reach the sample sizes stated in the methods.

#### Standard ASL acquisition

A flow-sensitive alternating inversion recovery (FAIR) labelling scheme was used for the acquisition of standard-ASL and BCSFB-ASL data [19, 31]. The FAIR technique alternates between a global inversion (non-selective, Mc) and a slice-selective inversion 32. The difference between the non-selective (labelled) and slice-selective (control) images results in a perfusion-weighted ASL image that reflects the signal from the labelled blood water that has moved into the imaging slice in the given inflow time (TI). Measuring this difference at multiple inflow times enables the quantification of perfusion related parameters, such as CBF. As described in previous work, FAIR-ASL data were acquired using a single slice, single shot spin echo – echo planar imaging readout, 20 mm slice-selective width, and a global labelling pulse (non-selective) [31].

Standard-ASL acquisition parameters: 1 mm imaging slice thickness, matrix size = 40 × 56, FOV = 20 mm × 20 mm, 7 dummy scans, TE = 20 ms. TI = [200, 500, 1000, 1500, 2000, 3000, 4000 ms], using a TR = 10,000 ms, 5 repetitions per TI [31].

Using the previously acquired sagittal anatomical reference images, the Standard-ASL imaging slice was manually positioned to align with the splenium of the corpus callosum in the mid-sagittal plane, in order to cover an area which includes parts of the cortex, hippocampus and midbrain.

#### BCSFB ASL acquisition

By using an ultra-long TE parameter in BCSFB-ASL, the acquisition is fine-tuned to detect the delivery of labelled arterial water across the BCSFB, into the ventricular CSF [19], due to the much longer T2 of CSF compared to parenchymal tissue. This BCSFB-ASL signal represents the total delivery of labelled water, and therefore does not provide a measure of CSF secretion, i.e. the net movement across the BCSFB. The BCSFB-ASL also employs a FAIR encoding with the same slice selective and global labelling pulses as for the standard ASL protocol.

BCSFB-ASL acquisition parameters: single slice, 2.4 mm imaging slice thickness, matrix size = 32 × 32, FOV = 20 mm × 20 mm, 5 dummy scans, TE = 220 ms. TI = [200, 200, 750, 1500, 2750, 4000, 5000, 6000 ms], using a recovery time (TRec) = 12,000 ms, 10 repetitions per TI [21, 31].

Using the previously acquired anatomical reference images, the BCSFB-ASL imaging slice was manually positioned to align with the caudal end of the lateral ventricles, as it has been shown to be densely populated with CP tissue [32]. The evaluation of BCSFB function in this study was centred on CP tissue in the lateral ventricles, excluding the 3rd and 4th ventricles.

### ASL-MRI analysis

**Fig. 1:**
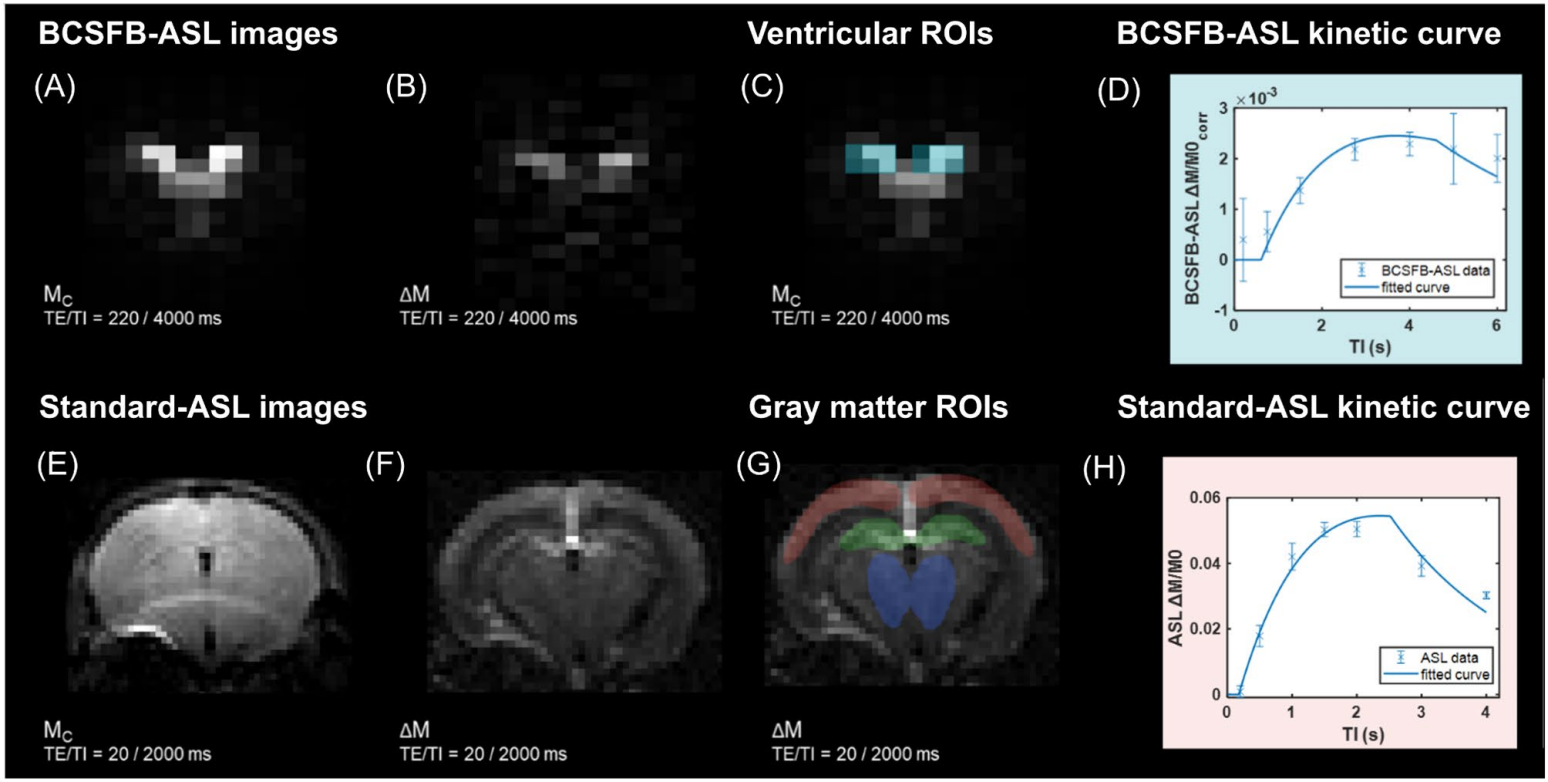
**Overview of the ASL images and contrasts**. (**A**)-(**B**) FAIR-ASL control (M_c_) and subtracted (ΔM) images for BCSBF-ASL (TE = 220 ms, TI = 4000 ms). (**C**) BCSFB-ASL ROI selection (turquoise) for BCSFB-mediated water delivery quantification, i.e. 6 voxels in the lateral ventricles. (**D**) ASL data and fitted kinetic curves for BCSFB-ASL for the ventricular ROIs. (**E**)-(**F**) FAIR-ASL control (M_c_) and subtracted (ΔM) images for standard-ASL (TE = 20 ms, TI = 2000 ms). (**G**) standard-ASL ROI selection for CBF quantification in three gray matter regions: cortex (red), hippocampus (green), midbrain (blue). (**H**) ASL data and fitted kinetic curves for standard-ASL using a cortical ROI. Representative data from a 20w normal control mouse (*N* = 1, error bars: ± stdev computed over repetitions)

Standard-ASL and BCSFB-ASL data were analysed to obtain absolute values of tissue CBF (cortical, HC, MB) and total BCSFB-mediated water delivery, respectively, as described previously [19] (see Fig. 1). For the quantification of CBF from standard-ASL data, three regions of interest (ROI) were drawn for each subject across the cerebral cortex, the hippocampus, and midbrain using the perfusion-weighted, ΔM FAIR-ASL image (see Fig. 1f), and the mean voxel signal was calculated across each ROI. For each ASL image pair, the non-selective mean ROI value (M_c_) was subtracted from the slice-selective (labelled) mean ROI value to provide the perfusion-weighted signal, ΔM. Repeated measures of ΔM and M_c_ at each inflow time were averaged to provide [TI, ΔM] and [TI, M_c_] datasets.

The [TI, M_c_] data were used to estimate the subject-wise values of M0, T1_tissue_ (M_c_ data at TE = 20 ms) and T1_CSF_ (M_c_ data at TE = 220 ms) by fitting the simple inversion recovery (IR) curve described in Eq. 2:

2 $$\:Mc=M0\cdot\:\left(1-2\cdot\:\text{e}\text{x}\text{p}\left(-TI/{T1}_{tissue}\right)\right)$$

T1_CSF_ is calculated specifically using the M_c_ data acquired at TE = 220 ms (BCSFB-ASL) to ensure that the signal present in the functional images is captured only from CSF compartments which have a long T2. At this TE, the voxels will contain signal almost exclusively from the CSF, without partial volume contributions from brain tissues or blood, thereby enabling a more accurate quantification of T1_CSF_ [19, 31, 33].

The [TI, ΔM] data were then used to fit the relevant Buxton kinetic model: a single-compartment general Buxton kinetic model for CBF from standard-ASL data [34], and a 2-compartment adaptation of the Buxton model for extracting rates of BCSFB-mediated water delivery from BCSFB-ASL data [19, 31, 35, 36], as described in Additional File (A1). For the calculation of per-subject hemodynamic outputs, subject-wise T1 (T1_tissue_ for standard ASL or T1_CSF_ for BCSFB-ASL) and M0 (or M0_corr_ for BCSFB-ASL) values extracted from the IR fittings were used in the Buxton model as inputs when calculating CBF and BCSFB-mediated water delivery values for each subject [19].

To determine an overall measure of total lateral ventricular BCSFB-function, a summary measure of the BCSFB-ASL signal was obtained across the lateral ventricles, which permitted the measurement of the total amount of labelled arterial-blood-water delivery to ventricular CSF. Thus, for the BCSFB-ASL images, two 3 × 2 voxel ROIs (12 voxels in total, ROI volume = 11.25 mm^3^) were positioned on a non-selective (control) image, overlaid with the position of the lateral ventricles (Fig. 1c). The combined ROI average signals were subtracted in a pairwise fashion to provide ΔM values. The calculated M0 will be highly dependent on ventricle size due to partial volume effects in the low resolution ASL images. A subject-wise ventricular volume normalisation factor was applied to M0_CSF_ to provide a volume-normalised, equilibrium magnetization, M0_corr_ (Eq. 3), which accounts for the difference between the total ventricular volume (quantified from T2-weighted anatomical images) and the ventricular ROI volume (i.e. from the 12 voxels used for the ROI from the low-resolution functional data with a volume of 11.25 mm^3^). This normalisation step is necessary for the accurate quantification of the total amount of BCSFB-mediated water delivery, and does not require normalisation of the BCSFB-ASL signal or ventricular volume to brain size [19].

3 $$\:{M0}_{corr}={M0}_{CSF}\times\:\frac{Total\:ventricular\:volume}{Total\:ROI\:volume}$$

The outputs of the model fittings provided subject-wise quantitative values for CBF and T1_tissue_ (T1_cortex_, T1_HC_, T1_MB_ for standard-ASL images), and rates of BCSFB-mediated water delivery alongside T1_CSF_ (BCSFB-ASL images). We report the group average of the individually extracted values.

### Histology

Following MRI, histopathological analysis of *N* = 3 animals per group at each time point was used to confirm the specific AD pathology of this mouse model in terms of amyloid plaques and neurofibrillary tangle deposition. Mice were anaesthetised and transcardially perfused with phosphate buffer saline, pH 7.0 and with 4% paraformaldehyde (PFA) in accordance with protocols approved by the Champalimaud Animal Welfare & Ethical Review Body (ORBEA). Brains were collected and post-fixed for 24 h in 4% PFA. To maintain consistency of dissection and to facilitate analysis in our cohort of mice, brains were coronally sliced in register with the Allen Mouse Brain Atlas (See http://mouse.brain-map.org). Coronal slabs were paraffin embedded and serially sectioned at 4 μm. Bregma levels 0.145, − 2.055, and − 2.88 were selected for immunohistochemistry for Tau and Aβ [37]. Tissue sections were immunostained for Tau and Aβ using Novolink™ Polymer (Leica Biosystems, UK), detected with DAB and counterstained with Harris Hematoxylin. For Tau, primary antibody (anti-Phospho-Tau, clone AT8, Thermo Fisher, cat. MN1020) was used at 1:100 dilution overnight at 4ºc, after antigen retrieval with citrate buffer pH6 for 20 min at 98º c. For Aβ, primary antibody (anti-Beta-Amyloid, clone 6E10, Biolegend, cat. 803007) was used at 1:750 dilution overnight at 4ºc, after antigen retrieval with 80% formic acid for 10 min at room temperature. Brightfield images were acquired with a Zeiss AxioScan.Z1 fully automated slide scanner using the 20x objective lenses. Finally, sections were analysed for density and spatial distribution of AT8 positive neurons and plaque distribution.

### Statistical methods

GraphPad Prism 10 was used to conduct statistical testing. Statistical comparisons between control and 3xTg data, at each time point, were conducted using unpaired, 2-tailed, Mann-Whitney tests, after Shapiro Wilkes normality testing revealed that most parameters were not normally distributed. Given that Shapiro Wilkes normality testing revealed that the behavioural data from the Y-maze (spontaneous alternations and total entries) was normally distributed, Welch’s t-tests were employed. Importantly, for the MR imaging data, and then separately for the behavioural data, the corrected false discovery rate (FDR) [38] method was used to account for multiple comparisons, with a desired FDR of 5%.

A two-way ANOVA was performed to investigate the effects of ageing, genetic background (control vs. AD), and their interaction, on BCSFB function. This was then followed by Sidak’s multiple comparisons correction.

We report the group mean of the data at a given time point, alongside the standard error of the mean (SEM). Overlaid onto the figures, asterisks denote the level of statistical significance achieved in our analyses: (*) represents significance at the *P* ≤ 0.05 level, (**) at *P* ≤ 0.01, (***) at *P* ≤ 0.001, and (****) at *P* ≤ 0.0001.

### Ex-vivo microimaging of choroid plexus

To compare the volume of the choroid plexus between control and AD mice, in a separate cohort of animals (N_NC_ = 3, mean age 12.5 months and N_TG_ = 4, mean age 14 months), we performed ultra-high resolution MRI of ex-vivo brain tissue, which enabled the direct imaging of the CP. The details regarding the sample preparation, image acquisition and data analysis for CP volume quantification are presented in the Additional File A5.

## Results

### ASL results

#### Standard ASL

**Fig. 2:**
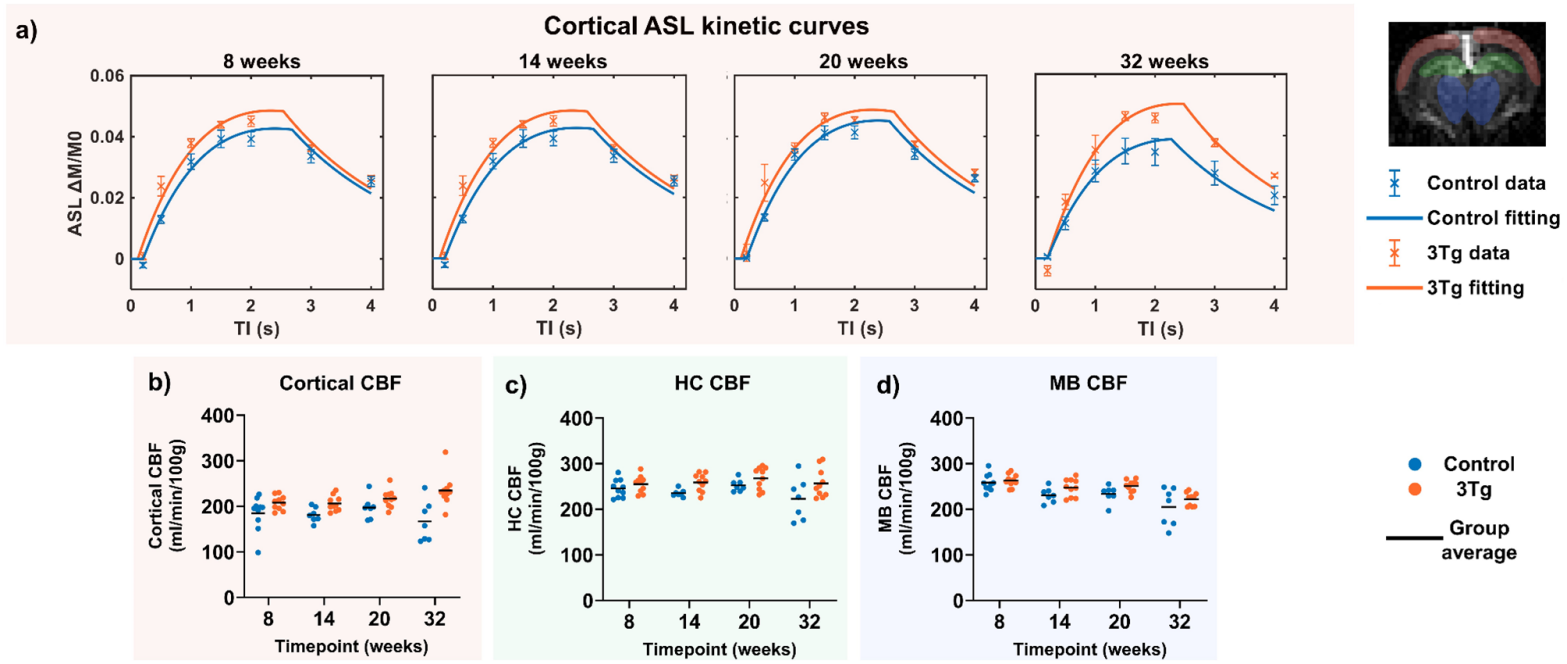
**Gray matter perfusion**. Standard-ASL MRI data describing blood flow. (**A**) Group-averaged cortical ASL kinetic curves for control and 3xTg cohorts. (**B**)-(**D**) individual subject and group averaged CBF values, at each time point for ROIs covering the cortex, hippocampus and midbrain

Standard-ASL data was acquired for the quantification of CBF in three gray matter regions: the cortex, hippocampus and midbrain (Fig. 2, Additional Fig. 1). For the cortical ROI, Fig. 2a presents the group-averaged kinetic curves, calculated by averaging ΔM/M0 across animals, which reveal slightly higher values in cortical CBF within the 3xTg group relative to the control group at all four timepoints. This increase in cortical CBF is most evident at 32 weeks. However, the comparison of individually extracted CBF values in all three ROIs (cortex, HC and MB) revealed no significant difference between the two groups at any time point, after adjusting for multiple comparisons, as illustrated in Fig. 2b-d and Table 1a.

**Table 1:** **ASL results.** (A) Summary of CBF values in the three ROIs calculated from standard ASL measurements for the different animal groups. The indicated p values are corrected for multiple comparisons. (B) Summary of BCSFB-ASL parameter values for the different animal groups. The reported p values are corrected for multiple comparisons

ROI	8 weeks			14 weeks			20 weeks			32 weeks		
	Ctrl	3xTg	*p*	Ctrl	3xTg	*p*	Ctrl	3xTg	*p*	Ctrl	3xTg	*p*
**(A)**	**CBF (ml/min/100 g)**											
Cortex	185 ± 11	208 ± 5	0.92	181 ± 6	206 ± 5	0.17	197 ± 9	217 ± 6	0.60	167 ± 17	235 ± 11	0.17
HC	246 ± 6	255 ± 6	> 0.99	235 ± 3	259 ± 6	0.17	252 ± 5	267 ± 8	> 0.99	222 ± 17	257 ± 10	> 0.99
MB	258 ± 6	263 ± 4	> 0.99	231 ± 6	247 ± 6	0.86	234 ± 7	251 ± 4	0.55	205 ± 16	222 ± 5	> 0.99
**(B)**	**BCSFB ASL parameters**											
Total water delivery (µl/min)	0.55 ± 0.09	1.13 ± 0.10	**0.036**	0.80 ± 0.07	1.34 ± 0.04	**0.015**	0.81 ± 0.06	1.39 ± 0.09	**0.036**	0.74 ± 0.11	1.32 ± 0.09	**0.041**
T1_CSF_ (s)	3.31 ± 0.13	3.64 ± 0.03	0.60	3.38 ± 0.17	3.65 ± 0.08	> 0.99	3.81 ± 0.04	3.89 ± 0.04	> 0.99	3.73 ± 0.19	3.81 ± 0.08	> 0.99
Ventricular volume (mm^3^)	1.02 ± 0.19	1.60 ± 0.11	0.55	1.52 ± 0.21	1.59 ± 0.10	> 0.99	1.83 ± 0.09	1.82 ± 0.04	> 0.99	1.89 ± 0.29	1.72 ± 0.12	> 0.99

T1 measurements in these regions also indicated that any differences between control and 3xTg groups were not statistically significant at any of the time points, after correcting for multiple comparisons. The data is presented in Supporting Information (Additional Fig. 1).

#### BCSFB ASL

**Fig. 3:**
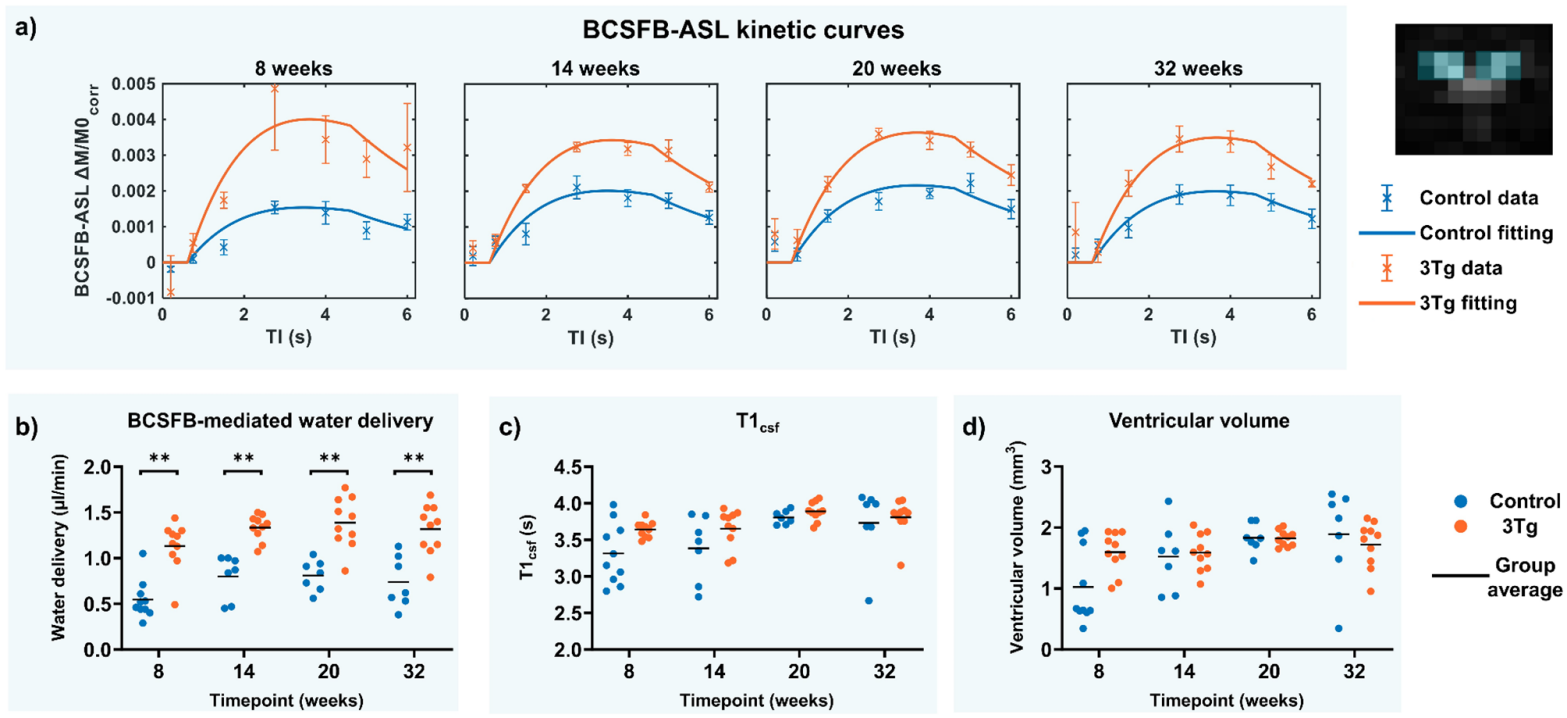
**BCSFB function and ventricular homeostasis**. BCSFB-ASL MRI data describing water delivery. (**A**) group-averaged kinetic curves for control and 3xTg cohorts, and (**B**) individual subject and group average total water delivery values, at each time point. (**C**) Individual subject and group average T1_CSF_ values from control image (Mc) data, and (**D**) ventricular volume measurements from structural MR data

BCSFB-ASL MRI data was collected to quantify total BCSBF-mediated water delivery (Fig. 3). Figure 3a presents the group-averaged kinetic curves, calculated by averaging ΔM/M0_corr_ across animals, which reveal increased water delivery in the 3xTg cohort, relative to strain-matched controls, at all four timepoints, an effect which can be readily observed from visual inspection of the kinetic curves (Fig. 3a). Figure 3b-d and Table 1b present the values of the estimated BCSFB-ASL parameters for different animal groups.

The BCSFB-mediated water delivery was significantly higher in the 3xTg animals compared to controls for all ages, while T1_CSF_ and ventricular volumes were not found to be significantly different between the groups, at any time point when correcting for multiple comparisons. T1_CSF_ at 8 and 14 weeks, and ventricular volumes at 8 weeks, displayed slightly higher values in the 3xTg mice, significant at the individual group level, but not after multiple comparisons correction.

The two-way ANOVA results summarised in Table 2 show that differences in BCSFB-mediated water delivery between control and AD subjects were significantly driven by the genetic background, i.e. control or AD status (*F*(1, 63) = [99.07], *P* < 0.0001), as well as by the age/time point (*F* = 4.534, *P* = 0.0061), although with a smaller effect size. The interaction term between genetic background and age was not significant (*F* = 0.04245, *P* = 0.99). Sidak’s test for multiple comparisons found that differences in the mean water delivery rate was significantly different exclusively in instances where controls were compared to 3xTg subjects, and not within the groups, as shown in Supporting Information (Additional Table 1).

**Table 2:** **Summary of the two-way ANOVA results**. A two-way ANOVA was conducted to investigate the effects of ageing (time point), genetic background (control vs. 3xTg status), and their interaction, on BCSFB-mediated water delivery rates. SS: sum of squares, DF: degrees of freedom, MS: mean squares

Source of variation	SS (Type III)	DF	MS	F	*P* value
Interaction	0.007187	3	0.002396	F = 0.04245	*P* = 0.9882
Time point	0.7677	3	0.2559	F = 4.534	*P* = 0.0061
Genetic background	5.591	1	5.591	F = 99.07	*P* < 0.0001
Residual	3.555	63	0.05643		
Total	9.920887	70			

### Behaviour

**Fig. 4:**
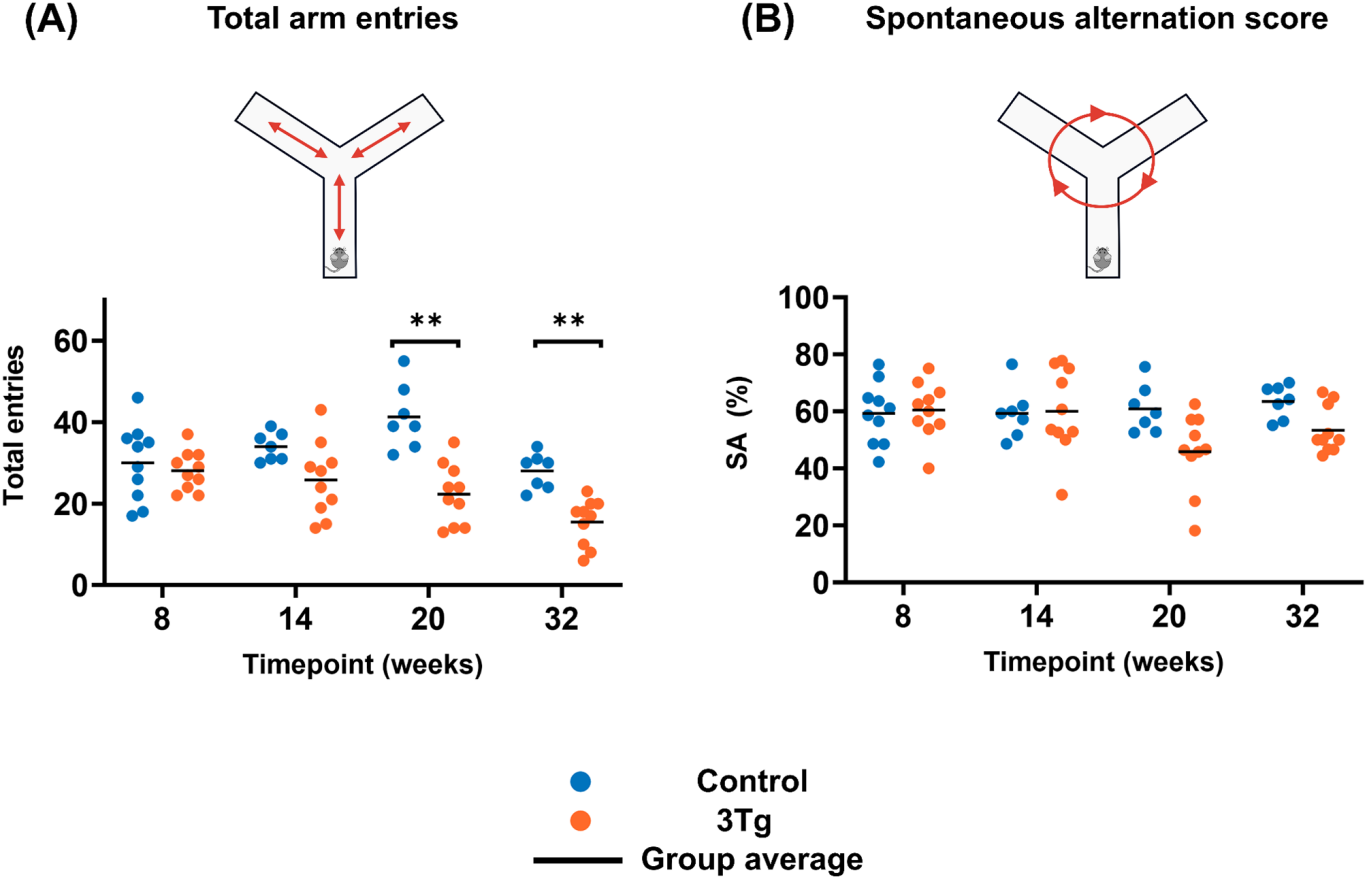
**Y maze behaviour analysis**. (**A**) total number of arm entries for control and 3xTg animals at different ages; (**B**) spontaneous alternation score for control and 3xTg animals at different ages

The behaviour assessment using the spontaneous alternations task showed significant difference between the control and the 3xTg mice starting with 20 weeks of age, as illustrated in Fig. 4. In particular, the number of arm entries is significantly reduced for the 3xTg animals (20w: [(control: 41 ± 8, 3xTg: 22 ± 7), *P* < 0.001], 32w: [(control: 28 ± 4, 3xTg: 16 ± 6), *P* < 0.001], Fig. 4a), while the SA score differences are not statistically significant when correcting for multiple comparisons (20w: [(control: 61 ± 8, 3xTg: 45 ± 14), uncorrected *P* = 0.019], 32w: [(control: 63 ± 6, 3xTg: 53 ± 8), uncorrected *P* = 0.013], Fig. 4b). Generally, the AD mice spent more time close to the middle of the maze, especially the older animals. We also observed a positive correlation between the SA score and the number of arm entries *(r* = 0.39, *P* = 0.01) for the 3xTg mice, while no such correlation was observed for the control animals. These behaviour results which probe the willingness of the mice to explore the maze, as well as their working memory, substantiate the known traits of the 3xTg mice which start to exhibit cognitive decline between 3- to 5- months of age [39].

### Histology

**Fig. 5:**
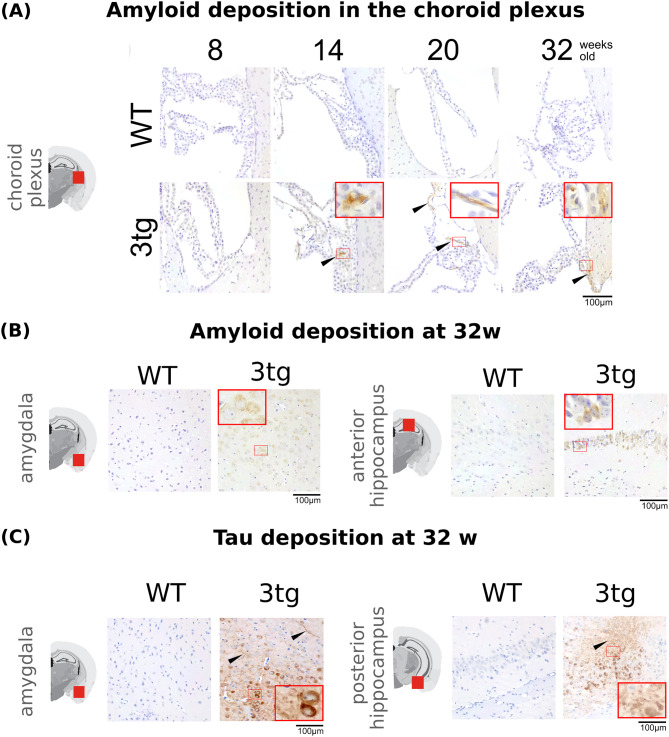
**Histological confirmation of AD pathology**. Immunohistochemical staining of Aβ and tau proteins in different areas of the brain for controls and 3xTg mice. (**A**) Aβ staining in the choroid plexus with plaques detected starting with 14 weeks. (**B**) Aβ staining in the hippocampus and amygdala at 32 weeks, showing wide-spread plaque formation. (**C**) Tau staining in the hippocampus and amygdala at 32 weeks, showing intracellular deposition and neurofibrillary tau tangles (black arrowhead).

Immunohistochemical staining of Aβ and tau proteins confirms the expected pathology of the 3xTg mouse model of AD (Fig. 5), with amyloid plaques detected in the choroid plexus (at the given slice location) starting at 14 weeks of age (Fig. 5a). We observe positive staining in regions of the brain involved early in the progression of AD, such as the hippocampus and amygdala, already at 8 weeks (Additional Fig. 2), with an increase in the staining intensity with age for both proteins. We also detect neurofibrillary tau-tangles starting at 20 weeks in the hippocampus and 32 weeks in the amygdala (Additional Fig. 3). As expected, no staining is observed in the control animals.

### Ex-vivo microimaging of choroid plexus

**Fig. 6:**
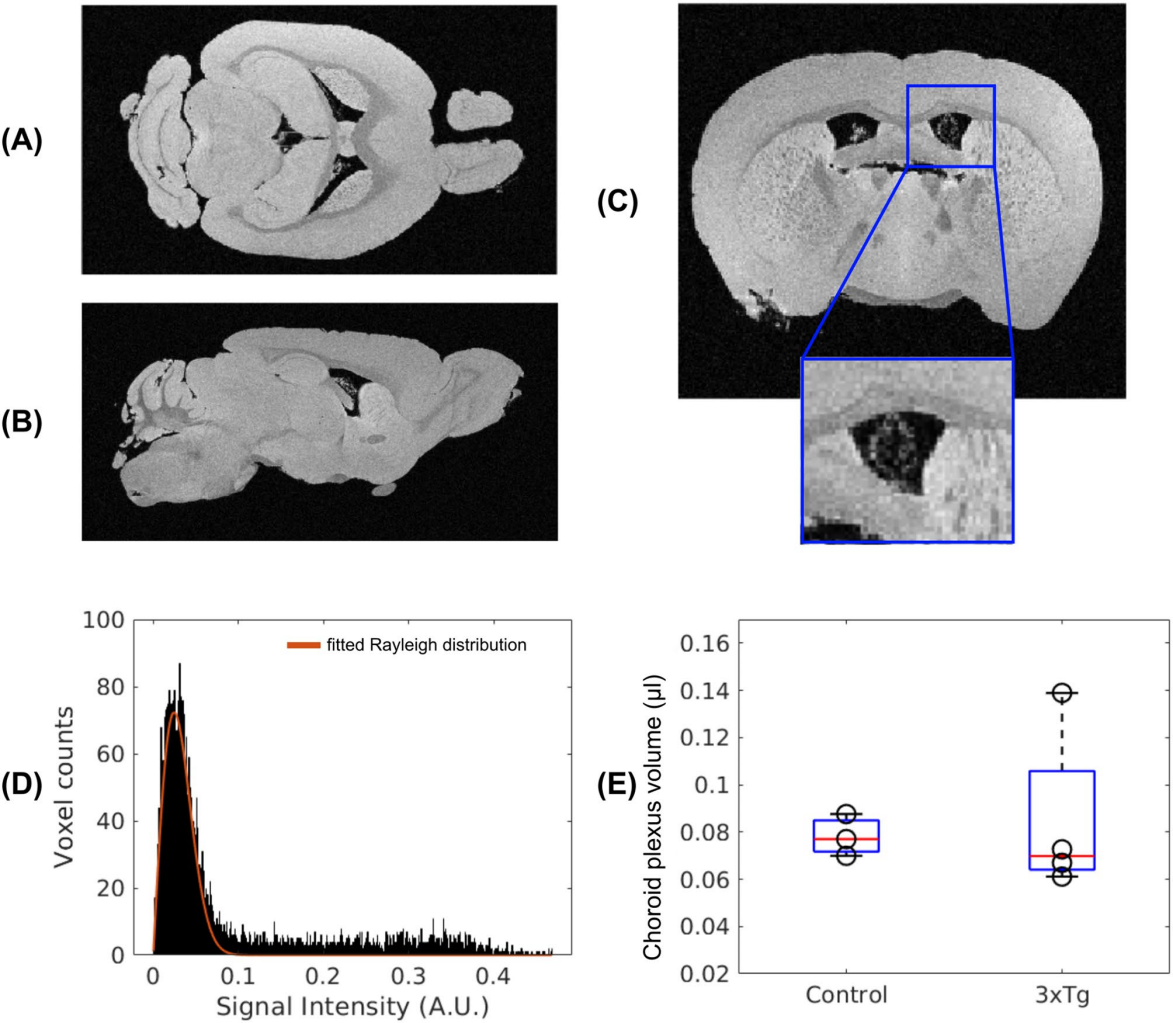
**Ex-vivo microimaging of CP.** (**A**)-(**C**) Example of MR microscopy images for one control mouse in the horizontal, sagittal and coronal planes, with an inset showing the clear contrast of the choroid plexus which appears as bright voxels inside the ventricles. (**D**) Distribution of signal intensity values inside the ventricles and fitted Rayleigh distribution. (**E**) Boxplots showing the median and interquartile range of CP volume for the controls and 3xTg animals

Figure 6a)-c) presents the MR microscopy images for a representative sample, where the CP is clearly visible as the high intensity tissue within the lateral ventricles. The difference between the voxel intensity values inside the ventricles and the Rayleigh distribution of noise is illustrated in Fig. 6d) and is then used to calculate the CP volume, as described in the Additional File A5. Figure 6e) shows the CP volumes estimated for different animals, with a mean value of 0.0782 ± 0.0089 µl for the control group and 0.0849 ± 0.0363 µl for the AD group. We do not observe significant differences between the normal controls and AD mice.

## Discussion

Our work harnessed a recently developed BCSFB-ASL MRI technique and showed, in-vivo, that CP function is altered in the early stages of Alzheimer’s disease in the widely employed triple transgenic mouse model. The measured differences in BCSFB function precede measured changes in behaviour or widespread deposition of neurofibrillary tangles, and are not accompanied by significant changes in ventricular volume or brain tissue perfusion, suggesting that, at least in this mouse model, the CP may play an important role in the early pathophysiology of AD. Since the data was found to not be normally distributed, comparisons between groups at each timepoint were conducted using Mann-Whitney tests, and then corrected for multiple comparisons. Given the non-invasive and translational nature of ASL MRI, measurements of BCSFB function can thus become a potential biomarker for AD.

### BCSFB-mediated water delivery

In this work we find that the 3xTg mouse model of AD consistently exhibits an elevated degree of BCSFB-mediated labelled blood-water delivery to the ventricular CSF (Fig. 3) relative to age-matched control mice. The ultra-long TE ASL measurement employed here reflects the exchange of the labelled blood water across the BCSFB, depending on the total volume of the CP in the LV convolved with its perfusion and the extraction fraction, i.e. the permeability of the BCSFB to labelled blood water. In order to further understand the observed increased water delivery in 3xTg mice, we performed ultra-high resolution ex-vivo MRI on perfused whole brain samples to measure the volume of the CP within the LV of 3xTg and control animals at 12–14 months of age. The results showed no significant difference in the estimated CPs volume between the groups. This finding, together with the fact that no significant differences in ventricular volume were observed between groups, suggest that a putative difference in CP or LV volumes do not underpin the observed increase in BCSFB-mediated water delivery in this animal model and that this is therefore reflective of a functional change at the choroid plexus. Moreover, we did not observe any correlation between the water delivery across BCSFB and the time of the day when the images were acquired.

We speculate that the increased rate of blood-to-CSF water delivery likely has two components: (1) a breakdown in the BCSFB integrity, giving rise to increased water permeability in the 3xTg model, due to the many morphological changes within CP tissue [4, 7], including the deposition of amyloid beta plaques (reported also in Fig. 5) and a loss of tight junction proteins previously reported in the literature [40]; (2) an increase in CP perfusion for boosting clearance due to the presence of adherent proteins. Such an increase in CP perfusion may be related to increased CSF secretion as a compensatory mechanism to increase CSF-mediated brain clearance [41]. This component also is consistent with recent observations in AD patients where increased perfusion to the CP was reported in AD patients relative to controls using standard ASL techniques [14]. Comparing the MRI metrics with the behavioural analysis did not reveal any statistically significant correlations.

### Current results in the context of known CP morphology and functional changes in AD

Investigations into CP tissue during AD in rodents have revealed that there is an impairment in function and metabolism of the BCSFB, with studies demonstrating that both the rate of secretion and the secretory profile of CSF are anomalous in the human and rodent brain during ageing and Alzheimer’s disease [4, 7, 40, 42–45]. In this work, histological analysis revealed the presence of amyloid in the CP tissue of 3xTg mice, supporting previous studies reporting on the role of CP in the clearance of amyloid beta from the CNS [3, 45]. For instance, previous histological analysis of mice injected with oligomeric Aβ revealed several proteins constituting cytoplasmic aggregates, including tau and amyloid. These aggregates not only mechanically disrupt plasma membranes of choroid plexus epithelial cells (CPecs), but also facilitate a marked decline in the integrity of the BCSFB in AD through the downstream activation of matrix metalloproteases, and a downregulation of enzymatic activity and tight junction (TJ) protein expression at the BCSFB locus [46]. Downregulation of genes related to TJ and a decrease in TJ protein levels have also been reported in human AD studies [43, 47]. These functional changes within the CPec working unit of the BCSFB will be closely intertwined with changes in CPec morphometry. Metabolic alterations [40], alongside oxidative stress – an early event in AD pathogenesis [48] – will lead to substantial CPec death. Together, these factors will likely have a drastic effect on the transport of water across the BCSFB, pointing to an increase in permeability of the BCSFB via (e.g.) paracellular means [43, 46, 47]. However, given the limited literature describing the effects of AD on the CP tissue and associated vasculature, as well as the fact that several functional parameters contribute to the BCSFB-ASL measurements, we cannot exclude the possibility of other factors increasing the measured rate of water delivery across the BCSFB in 3xTg mice, such as increased activity of transporters involved in water movement, as well as increased perfusion to the BCSFB itself.

### Tissue perfusion

A recent meta-analysis of neuroimaging data [49] found ASL MRI measures of cerebral perfusion to show the greatest degree of ‘biomarker abnormality’ at the early stages of late onset Alzheimer’s Disease. Thus, in the present study we hypothesised that ASL measures of brain tissue perfusion (here taken in the cortex, hippocampus and midbrain) would differ between the 3xTg model and controls. However, although higher mean CBF in the 3xTg was observed at selected timepoints (see Fig. 2), when accounting for multiple comparisons we found no significant differences between the 3xTg and control groups in our ASL measures of tissue perfusion within the ROIs examined across the timepoints. To our knowledge this is the first report of cerebral perfusion measurements in the 3xTg model. This observation is broadly consistent with previous findings [50] where no difference in whole brain uptake of 18 F-FDG was found between 11-month 3xTg and control mice. However, other studies report hypometabolism of 18 F-FDG in the 16 month 3xTg [51] which may be expected to be accompanied by a concomitant reduction in perfusion as assessed with ASL-MRI.

The 3xTg model does not exhibit overt deposition of amyloid beta in the cerebral arteries and as such presents as a limited model of cerebral amyloid angiopathy (CAA) [52]. This may be a key reason why no differences in cortical perfusion were detected in the 3xTg model vs. the control, aged-matched mice. Clinically, the presence of CAA is likely to have implications for CSF physiology as this may impair the function of the putative glymphatic system which operates via the traversal of CSF to and from the tissue via perivascular channels. This in turn may influence CSF secretion at the CP though perturbations of downstream CSF-ISF exchange pathways.

### Ageing effects on water delivery across BCSFB

Post-hoc analyses were conducted to determine the extent to which subject age was playing a role in the differences observed in our hemodynamic biomarkers. From the two-way ANOVA, both the effect of genetic background and ageing were shown to be significant drivers, with no significant interaction term. Following multiple comparison corrections, significant differences in BCSFB-mediated water delivery were observed only when comparing control with 3xTg groups at any time point, and not when comparing animals with the same genetic background but different ages. Thus, our findings suggest that the underlying AD pathology is indeed the primary driver for the observed changes in BCSFB water delivery, regardless of the specific time point, with significant differences observed already at 8 weeks of age, corresponding to the presymptomatic stage of the disease. This finding is consistent with a recent proteomic study reporting significant CP tissue differences between AD and control mice already at 7 weeks [53]. To study the effect of aging and pathology progression, a larger number of animals with a wider range of ages and/or a longitudinal study are needed.

### Methodological perspectives

In this work, the BCSBF-ASL methodology has been applied on a Bruker 9.4T MR system, in combination with a cryogenic receive coil to study the BCSFB in the mouse brain with high SNR. Nevertheless, the BCSBF-ASL methodology has been successfully applied both on a 3.0 T clinical scanner [22] as well as 9.4 T preclinical MR scanners (e.g [19, 20]). using a more standard (i.e. non-cryogenic) receiver coil. The versatility of this methodology is an important aspect to consider for its widespread use in future studies, as this contrast is dependent on the selection of appropriate MR parameters, namely the ultra-long TE to achieve an isolation of the signal in the CSF, as opposed to specific hardware or software requirements.

### Limitations and future work

There are several limitations to this study. For example, this work clearly demonstrates a derangement in BCSFB function in the 3xTg mouse model of AD compared to age-matched controls. However, it is important to note that the BCSFB-ASL measurements reflect the convolution of the total CP volume with its perfusion and the extraction fraction. To disentangle these effects, future studies could also include measurements of CP perfusion, which are highly challenging due to the required resolution. Nevertheless, several steps have been taken in this direction both in mice [54] and in humans [55] by acquiring ASL data with high spatial resolution and/or multiple echo times. We can further leverage an innovative bed design for acquiring high resolution ASL data in rodents that we recently proposed [56], as well as denoising strategies for the analysis [57].

MRI measurements were non-invasively collected in the anaesthetised mouse brain, but not the awake brain. Anaesthetic protocols are known to variably affect cerebral hemodynamics in rodent strains, which can limit the accuracy of quantification of hemodynamic parameters such as CBF [58, 59] relative to the ground truth under normal awake physiology. However, awake imaging is highly challenging in mice, with further concerns regarding the impact of stress on hemodynamic markers [60, 61]. Thus, the use of a standardised protocol which prioritises high scan-rescan reproducibility [19], fast reversibility and recovery [59, 62], as well as straightforward administration, was favourable in this work, nevertheless other protocol could be explored in future work.

Another limiting factor is the cross-sectional study design including only female mice. We are unable to evaluate subject-wise longitudinal changes or the effect of gender on the hemodynamic biomarkers. A longitudinal study design including even younger animals could be considered in future work to provide further aetiological understanding of the disease onset. Here we chose to include only females due to ethics concerns raised by aggressive behaviour leading to increased mortality in male mice, in line with the experience of other groups [63, 64].

The scope of the histological analysis in this study was mainly focused on confirming the presence of known pathology, in terms of the deposition of amyloid plaques and neurofibrillary tangles. The limited sample size (*N* = 3 per group, per time point) and spatial coverage are insufficient for quantitative comparisons between the in-vivo biomarkers and brain-wide burden of plaques and tangles. Future work could also examine the TJ proteins to provide insights into whether the increased water accumulation in the CP is due to disruptions in barrier integrity. Such experiments could complement MRI findings by confirming if structural changes at the molecular level correlate with the functional changes observed, thereby providing further insight into the underlying mechanistic changes that lead to the observed differences in BCSFB-mediated water delivery.

## Conclusion

Contributing to the recent efforts in understanding the critical role of the CP-BCSFB locus in AD pathology, this study shows a significant increase in BCSFB-mediated water delivery in the 3xTg mouse model of AD, for the first time. Our findings are also in line with recent clinical studies showing an increase in apparent CP perfusion in AD [14, 65]. The far greater effect size of the BCSFB-ASL measurements relative to traditional ASL measurements suggests that the BCSFB-ASL method may provide a potential, readily translatable biomarker of early AD pathology.

## Electronic supplementary material

Below is the link to the electronic supplementary material.

Supplementary Material 1: The additional file consists of five sections, presenting a detailed description of the ASL modelling approaches (A1), T1 values across different brain regions (A2), the post-hoc comparison of BCSFB water delivery across different groups (A3), additional histology plots for animals of different ages (A4), as well as a detailed description of the methodology for the CP quantification based on ex-vivo microimaging (A5).

## Data Availability

The datasets used and/or analysed during the current study are available from the corresponding author on reasonable request. Any requests for the data and code should be submitted to: andrada.ianus@kcl.ac.uk and/or c.perera@ucl.ac.uk.

## Abbreviations

CP: Choroid plexus
BCSFB: Blood-cerebrospinal fluid-barrier
AD: Alzheimer’s disease
CBF: Cerebral blood flow
3xTg: Triple transgenic mouse model
CSF: Cerebrospinal fluid
ASL: Arterial spin labelling
DCE: Dynamic contrast enhanced
Aβ: Amyloid beta
NC: Normal control
SA: Spontaneous alternations
%SA: SA score
HC: Hippocampus
MB: Midbrain
FOV: Field of view
TE: Echo time
TR: Repetition time
TI: Inflow time
TRec: Recovery time
ROI: Region of interest
SS: Sum of squares
DF: Degrees of freedom
MS: Mean squares
TJ: Tight junctions
