# Supplementary material for: Non-invasive MRI of blood-cerebrospinal fluid-barrier function in a mouse model of Alzheimer’s disease: a potential biomarker of early pathology
Source: Fluids Barriers CNS. 2024 Dec 4;21:97. doi: 10.1186/s12987-024-00597-7 (PMC11616325; doi:10.1186/s12987-024-00597-7)
Supplement: Supplementary file 1 — Supplementary Material 1: The additional file consists of five sections, presenting a detailed description of the ASL modelling approaches (A1), T1 values across different brain regions (A2), the post-hoc comparison of BCSFB water delivery across different groups (A3), additional histology plots for animals of different ages (A4), as well as a detailed description of the methodology for the CP quantification based on ex-vivo microimaging (A5). [file 12987_2024_597_MOESM1_ESM.pdf]

Non-invasive MRI of Blood-Cerebrospinal Fluid-Barrier Function: a Potential Biomarker of Early Alzheimer's Disease Pathology

The additional file consists of five sections, presenting a detailed description of the ASL modelling approaches (A1), T1 values across different brain regions (A2), the post-hoc comparison of BCSFB water delivery across different groups (A3), additional histology plots for animals of different ages (A4), as well as a detailed description of the methodology for the CP quantification based on ex-vivo microimaging (A5).

A1 – ASL modelling

Standard ASL quantification

For standard ASL, cerebral blood flow (CBF) for the cortex, HC, and MB, was quantified by taking the ASL signal, i.e. ΔM values at TE = 20 ms, and fitting the general kinetic Buxton model (single compartment model), as described by equation 3 from Buxton et al 1998 (1).

BCSFB-ASL quantification

*R*ates of delivery of labelled blood water to ventricular CSF from BCSFB-ASL images (TE = 220 ms) were quantified using the 2-compartment perfusion model. This 2-compartment model was first described by Alsop and Detre (2), then later adapted by Wong et al. (3), and then implemented by Wang et al. (4). Subsequently, this model was further adapted by Evans et al. for the purposes of describing the transfer of labelled water from blood into the ventricular CSF, rather than into the cerebral cortex (5), based on data acquired at ultra-long echo time, and is given by the equations below:

TI range: (0 > TI > Δt)

$$\Delta M = 0$$

TI range: (TI > Δt)

$$\Delta M = \frac{2.M_0.f.\alpha}{\varphi}\{exp(-TI\,.R_1).[exp(min(TI,\Delta t+\tau).\Delta R)-exp(\Delta t.\Delta R)]$$
$$-\,exp\,(\Delta t.\Delta R)]/\Delta R]\}$$

$$R_1 \;=\; {}^{1}\!/_{T1_{csf}}$$

$$\Delta R \;=\; \left(R_1+\frac{f}{\varphi}\right)-\left(\frac{1}{T1_b}\right)$$

Equation A1 - Adapted 2-compartment Buxton kinetic model for quantification of BCSFB-mediated water delivery rates from BCSFB-ASL data (ultra-long TE, 220 ms).

This 2-compartment model redefines the arrival time of the tagged bolus (Δt) to describe a transit time into the true tissue of interest, but also δ_a – the transit time for the labelled bolus to reach the imaging slice. By having these separate arrival times, the proximal artery signal is now modelled and accounted for, and labelled spins may enter the tissues before imaging. Thus, this model now directly accounts for the contribution of intraluminal/intravascular (IV) spins. The transit time of labelled blood water from the labelling slab to the vascular compartment of the imaging slice can be accounted for.

Further adaptations to the model have been made to include only the equation describing "extravascular signal", ΔM_{EV}, which applies to the measured BCSFB-ASL signal that derives from labelled water delivery to the CSF, i.e. the true "tissue" compartment. Here, an adjustment is made from the equations used in the work by Wang et al (4): the equation describing the signal contribution from intravascular (IV) spins, ΔM_{IV} (where TI < Δt, before the arrival of the labelled bolus into ventricular CSF), is excluded. At TI values before Δt, the labelled water would not have crossed the BCSFB, and this signal would describe water still within the vasculature. Due to use of an ultra-long echo time (220 ms), signal arising from labelled water in blood are nulled (6). Overall, this adaptation allows for the model to be utilised to describe the delivery of labelled blood water into the CSF compartment, as opposed to brain tissue in standard-ASL (Equation S1).

This 2-compartment model requires several inputs: $\Delta M/M0_{corr}$, $T1_{CSF}$, inversion efficiency (α = 0.9), and the longitudinal relaxation of arterial blood ($T1_b$ = 2.5 s, from the literature (7)). R1 is

the longitudinal relaxation rate of brain tissue in the absence of blood flow. Here, ϕ, defines the blood-to-CSF water partition coefficient (5), which is now the density of water in the blood divided by the density of water in the CSF. As such, φ is taken as 1 (5,8–10).

Curve Fitting Toolbox (Matlab) was used throughout this work to fit kinetic curves to the acquired multi-TI standard-ASL and BCSFB-ASL data to the models described, using in-house scripts (5,11–13).

A2 – Standard ASL across brain regions

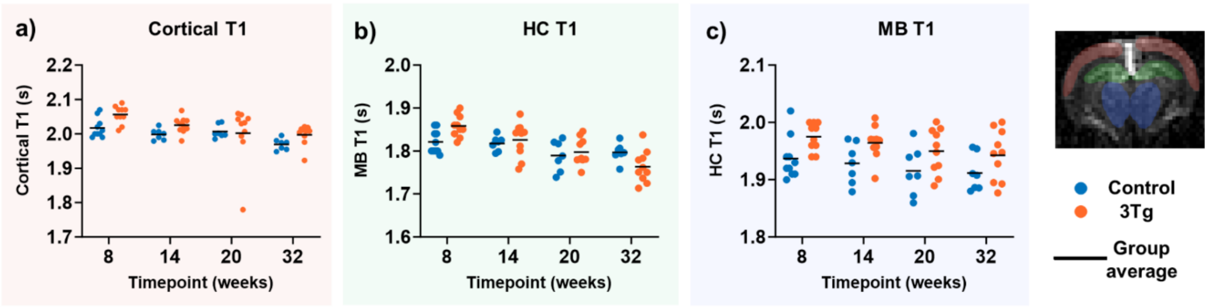

Figure A1. T1 in gray matter ROIs. Individual subject T1 values extracted from control image (Mc) standard-ASL data, at different time points, in a) the cortex, b) the hippocampus (HC), and c) the midbrain (MB).

A3 – Effects of age and genetic background on BCSFB water delivery

Following the Two-Way ANOVA to investigate the effects of ageing and genetic background on BCSBF function, we conducted post-hoc, Sidak's multiple comparisons testing. The results for different groups are presented in Table S1.

**Šídák's multiple comparisons test**	**Summary**	**Adjusted P Value**
**8w control vs. 8w 3xTg**	****	<0.0001

**8w control vs. 14w control**	ns	0.6108
**8w control vs. 14w 3xTg**	****	<0.0001
**8w control vs. 20w control**	ns	0.5254
**8w control vs. 20w 3xTg**	****	<0.0001
**8w control vs. 32w control**	ns	0.9562
**8w control vs. 32w 3xTg**	****	<0.0001
**8w 3xTg vs. 14w control**	ns	0.1549
**8w 3xTg vs. 14w 3xTg**	ns	0.8325
**8w 3xTg vs. 20w control**	ns	0.1977
**8w 3xTg vs. 20w 3xTg**	ns	0.4289
**8w 3xTg vs. 32w control**	*	0.0342
**8w 3xTg vs. 32w 3xTg**	ns	0.9246
**14w control vs. 14w 3xTg**	***	0.0007
**14w control vs. 20w control**	ns	>0.9999
**14w control vs. 20w 3xTg**	***	0.0001
**14w control vs. 32w control**	ns	>0.9999
**14w control vs. 32w 3xTg**	**	0.0011
**14w 3xTg vs. 20w control**	***	0.0009

**14w 3xTg vs. 20w 3xTg**	ns	>0.9999
**14w 3xTg vs. 32w control**	****	<0.0001
**14w 3xTg vs. 32w 3xTg**	ns	>0.9999
**20w control vs. 20w 3xTg**	***	0.0002
**20w control vs. 32w control**	ns	>0.9999
**20w control vs. 32w 3xTg**	**	0.0016
**20w 3xTg vs. 32w control**	****	<0.0001
**20w 3xTg vs. 32w 3xTg**	ns	>0.9999
**32w control vs. 32w 3xTg**	***	0.0002

Table A1: Post-hoc multiple comparisons results from two-way ANOVA presenting group differences in BCSBF-mediated water delivery.

A4 – Histology: Aβ and tau staining at different ages

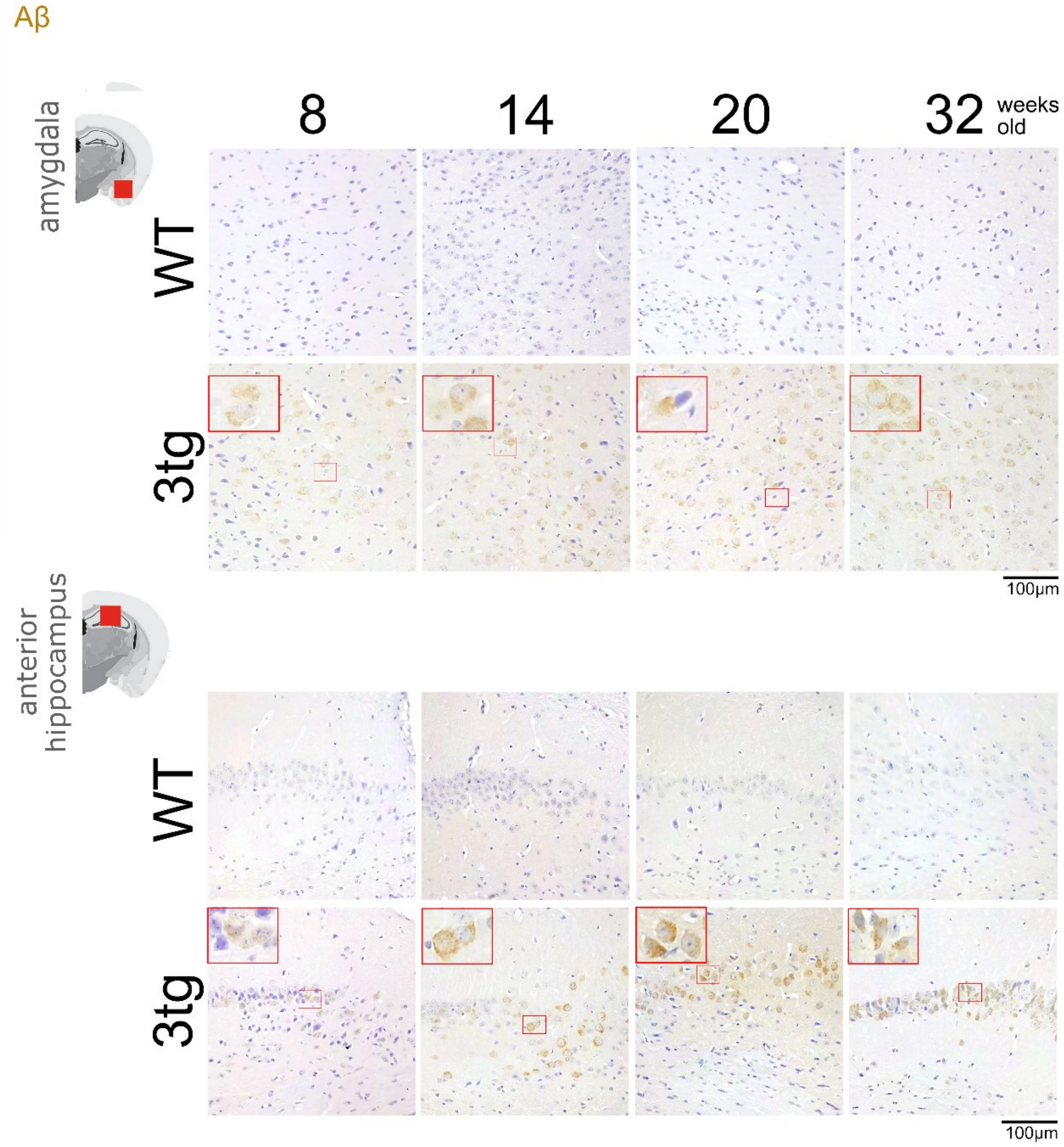

Figure A2. Immunohistochemical staining of amyloid beta in the anterior cortical (top) and hippocampal (bottom) regions for wild type and 3xTg mice of different ages. In the 3xTg animals, plaques are detected already ay 8 weeks, although the staining becomes more pronounced after 14 weeks. No plaques are detected in the control animals.

TAU

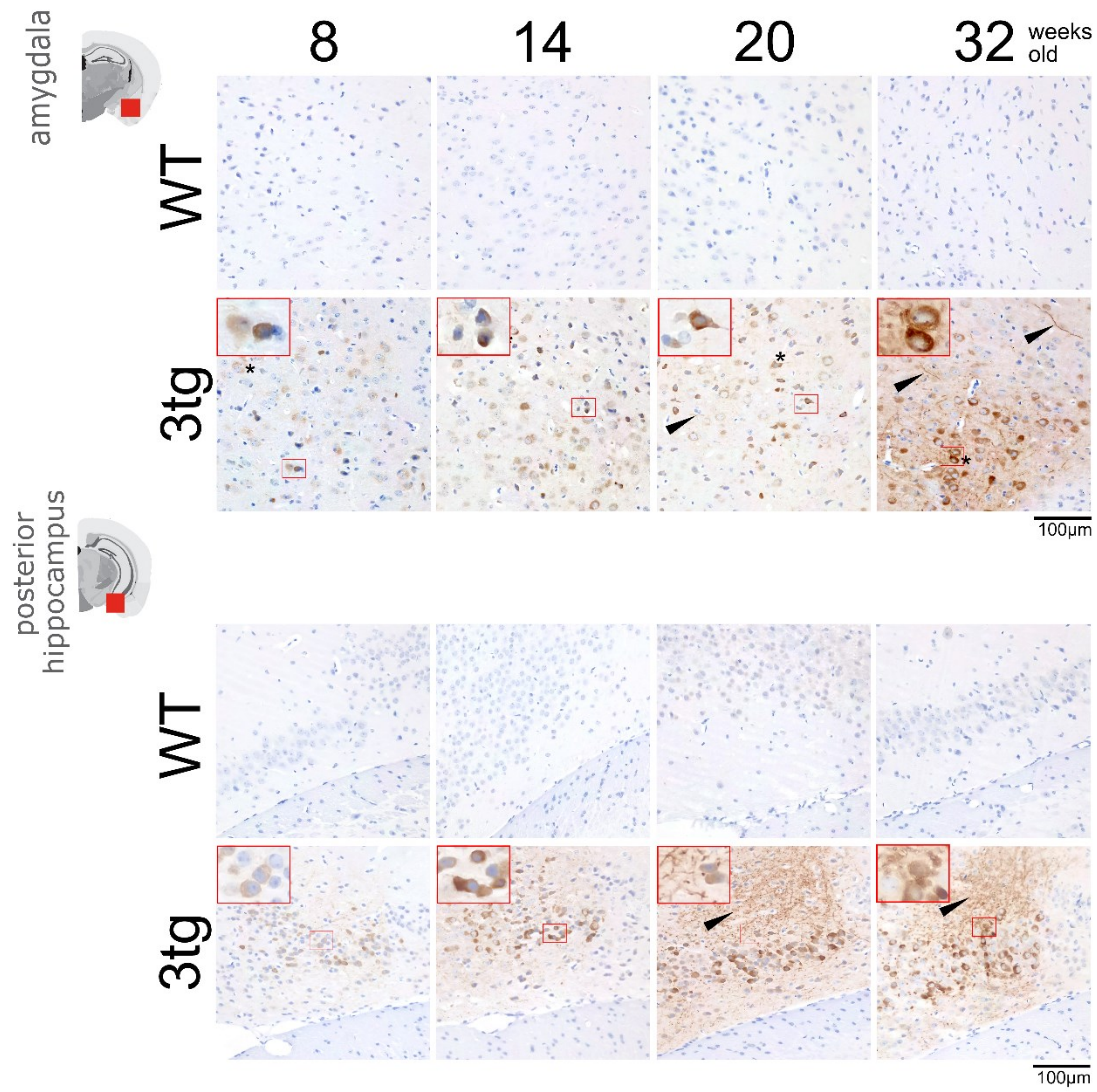

Figure A3. Immunohistochemical staining of tau in the anterior cortical (top) and posterior hippocampal (bottom) regions for wild type and 3xTg mice of different ages. In the 3xTg animals, positive tau stains are detected already ay 8 weeks, and axonal depositions of tau (arrowheads) are observed from 20 weeks. No tau staining is detected in the control animals.

A5 - Ex-vivo microimaging of choroid plexus

To compare the volume of the choroid plexus between control and AD mice, in a separate cohort of animals we performed ultra-high resolution MRI of ex-vivo brain tissue.S5

Sample preparation

For ex-vivo MRI, female mice (N_{NC} = 3, mean age 12.5 months and N_{TG} = 4, mean age 14 months) were anaesthetised and transcardially perfused with 4% PFA. The brains were surgically removed, post-fixed for 24 h in 4% PFA and rehydrated in PBS for at least 24h (14). Before being mounted in 10 mm NMR tubes filled with fluorinert, the brains were submerged in fluorinert and the cerebral hemispheres were slightly separated with a flexible spatula, so that fluorinert enters the ventricular system. In this way, we ensured an excellent contrast to directly image and quantify the volume of the choroid plexus.

Image acquisition

Ultra-high resolution data at 40 µm isotropic was acquired on a 16.4T Aeon Ascent Bruker Scanner equipped with a Micro 5 imaging probe capable of generating 3T/m gradients. Images were acquired using a 3D gradient echo (GE) sequence with the following parameters: TE/TR = 5/45 ms, matrix size = 450 x 225 x 255, flip angle = 12°, NA = 12 averages, with a total scan duration of ~8h per sample.

Data analysis

The volume of the choroid plexus was calculated from the histogram analysis of the ventricular signal. Following the manual delineation of the lateral ventricles, including the background signal and choroid plexuses, the histogram of the signals was analysed to quantify the volume of the choroid plexus while accounting for partial volume in the imaging voxels. First, the main peak of the distribution, which corresponds to the background signal (noise) in areas filled with fluorinert, was fitted with a Rayleigh distribution describing the MRI noise in the absence of signal. Next, to determine the tissue signal, the fitted Rayleigh distribution was subtracted from

the original histogram. The resulting histogram was rescaled between a lower signal boundary (LB) corresponding to a voxel with no tissue and an upper signal boundary (UB) corresponding to a voxel with 100% tissue. We calculated LB as the mean of the Rayleigh distribution, and UB as two noise standard deviations below the maximum measured signal. Then, to account for partial volume effects, the CP volume was calculated from the histogram count weighted by the signal value.

References

1. Buxton RB, Frank LR, Wong EC, Siewert B, Warach S, Edelman RR. A general kinetic model for quantitative perfusion imaging with arterial spin labeling. Magn Reson Med. 1998 Sep;40(3):383–96.

2. Alsop DC, Detre JA. Reduced transit-time sensitivity in noninvasive magnetic resonance imaging of human cerebral blood flow. J Cereb Blood Flow Metab. 1996 Nov;16(6):1236–49.

3. Wong EC, Buxton RB, Frank LR. A theoretical and experimental comparison of continuous and pulsed arterial spin labeling techniques for quantitative perfusion imaging. Magn Reson Med. 1998 Sep;40(3):348–55.

4. Wang J, Alsop DC, Li L, Listerud J, Gonzalez-At JB, Schnall MD, et al. Comparison of quantitative perfusion imaging using arterial spin labeling at 1.5 and 4.0 Tesla. Magn Reson Med. 2002 Aug;48(2):242–54.

5. Evans PG, Sokolska M, Alves A, Harrison IF, Ohene Y, Nahavandi P, et al. Non-Invasive MRI of Blood–Cerebrospinal Fluid Barrier Function. Nat Commun. 2020 Apr 29;11(1):1–11.

6. Ohene Y, Harrison IF, Nahavandi P, Ismail O, Bird EV, Ottersen OP, et al. Non-invasive MRI of brain clearance pathways using multiple echo time arterial spin labelling: an aquaporin-4 study. Neuroimage. 2019 Mar;188:515–23.

7. Dobre MC, Uğurbil K, Marjanska M. Determination of blood longitudinal relaxation time (T1) at high magnetic field strengths. Magn Reson Imaging. 2007 Jun;25(5):733–5.

8. Chappell MA, McConnell FAK, Golay X, Günther M, Hernandez-Tamames JA, van Osch MJ, et al. Partial volume correction in arterial spin labeling perfusion MRI: A method to disentangle anatomy from physiology or an analysis step too far? Neuroimage. 2021 Sep;238:118236.

9. Herscovitch P, Raichle ME. What is the correct value for the brain--blood partition coefficient for water? J Cereb Blood Flow Metab. 1985 Mar;5(1):65–9.

10. Choi JD, Moon Y, Kim H-J, Yim Y, Lee S, Moon W-J. Choroid Plexus Volume and Permeability at Brain MRI within the Alzheimer Disease Clinical Spectrum. Radiology. 2022 Sep;304(3):635–45.

11. Perera C, Harrison IF, Lythgoe MF, Thomas DL, Wells JA. Pharmacological MRI with Simultaneous Measurement of Cerebral Perfusion and Blood-Cerebrospinal Fluid Barrier Function using Interleaved Echo-Time Arterial Spin Labelling. Neuroimage. 2021 Sep;238:118270.

12. Perera C, Tolomeo D, Baker RR, Ohene Y, Korsak A, Lythgoe MF, et al. Investigating changes in blood-cerebrospinal fluid barrier function in a rat model of chronic hypertension using non-invasive magnetic resonance imaging. Front Mol Neurosci. 2022 Sep 2;15:964632.

13. Perera P. Development and Application of MRI Techniques for Non-invasive Assessment of Blood-cerebrospinal Fluid Barrier Function. UCL (University College London); 2023.

14. Schilling KG, Grussu F, Ianus A, Hansen B, Barrett RLC, Aggarwal M, et al. Recommendations and guidelines from the ISMRM Diffusion Study Group for preclinical diffusion MRI: Part 2 -- Ex vivo imaging [Internet]. arXiv [physics.med-ph]. 2022. Available from: http://arxiv.org/abs/2209.13371
